# Critical indirect effects of climate change on sub-Antarctic ecosystem functioning

**DOI:** 10.1002/ece3.678

**Published:** 2013-07-31

**Authors:** E Louise Allan, P William Froneman, Jonathan V Durgadoo, Christopher D McQuaid, Isabelle J Ansorge, Nicole B Richoux

**Affiliations:** 1 Department of Zoology and Entomology, Rhodes UniversitySouth Africa; 2 GEOMAR Helmholtz-Zentrum für Ozeanforschung KielGermany; 3 Oceanography Department, Marine Research Institute, University of Cape TownSouth Africa

**Keywords:** Climate change, food web, frontal shifts, land-based top predators, Prince Edward Islands, Southern Ocean, stable isotope signatures, sub-Antarctic Front

## Abstract

Sub-Antarctic islands represent critical breeding habitats for land-based top predators that dominate Southern Ocean food webs. Reproduction and molting incur high energetic demands that are sustained at the sub-Antarctic Prince Edward Islands (PEIs) by both inshore (phytoplankton blooms; “island mass effect”; autochthonous) and offshore (allochthonous) productivity. As the relative contributions of these sustenance pathways are, in turn, affected by oceanographic conditions around the PEIs, we address the consequences of climatically driven changes in the physical environment on this island ecosystem. We show that there has been a measurable long-term shift in the carbon isotope signatures of the benthos inhabiting the shallow shelf region of the PEIs, most likely reflecting a long-term decline in enhanced phytoplankton productivity at the islands in response to a climate-driven shift in the position of the sub-Antarctic Front. Our results indicate that regional climate change has affected the balance between allochthonous and autochthonous productivity at the PEIs. Over the last three decades, inshore-feeding top predators at the islands have shown a marked decrease in their population sizes. Conversely, population sizes of offshore-feeding predators that forage over great distances from the islands have remained stable or increased, with one exception. Population decline of predators that rely heavily on organisms inhabiting the inshore region strongly suggest changes in prey availability, which are likely driven by factors such as fisheries impacts on some prey populations and shifts in competitive interactions among predators. In addition to these local factors, our analysis indicates that changes in prey availability may also result indirectly through regional climate change effects on the islands' marine ecosystem. Most importantly, our results indicate that a fundamental shift in the balance between allochthonous and autochthonous trophic pathways within this island ecosystem may be detected throughout the food web, demonstrating that the most powerful effects of climate change on marine systems may be indirect.

## Introduction

The Antarctic Circumpolar Current (ACC) is a key feature of the Southern Ocean and a primary factor shaping Southern Ocean ecosystems (Tynan 1998). Recent investigations using coupled ocean-atmosphere climate models suggest that the westerly wind belt that drives the ACC is intensifying and shifting polewards in response to global climate change (Thompson and Solomon 2002; Oke and England 2004; Fyfe et al. 2007). These adjustments are mirrored by a gradual southward migration of the ACC (Fyfe and Saenko 2005; Sokolov and Rintoul 2009; Downes et al. 2011), which has seen a southward shift of up to ∼1° Lat since the 1950s (Gille 2002). The sub-Antarctic Prince Edward Islands (PEIs) lie in the direct path of the ACC and provide a critical habitat for up to five million top predators (pinnipeds, flying seabirds, and penguins) that breed on land (Williams et al. 1979; Condy 1981; Ryan and Bester 2008). The abundance and diversity of organisms associated with these islands confers on them elevated ecological and conservation status (Ryan and Bester 2008) and makes the PEIs ideal laboratories to study the sensitivity and adaptation of sub-polar ecosystems to long-term natural and anthropogenic changes (Smith 2002).

**Figure 1:**
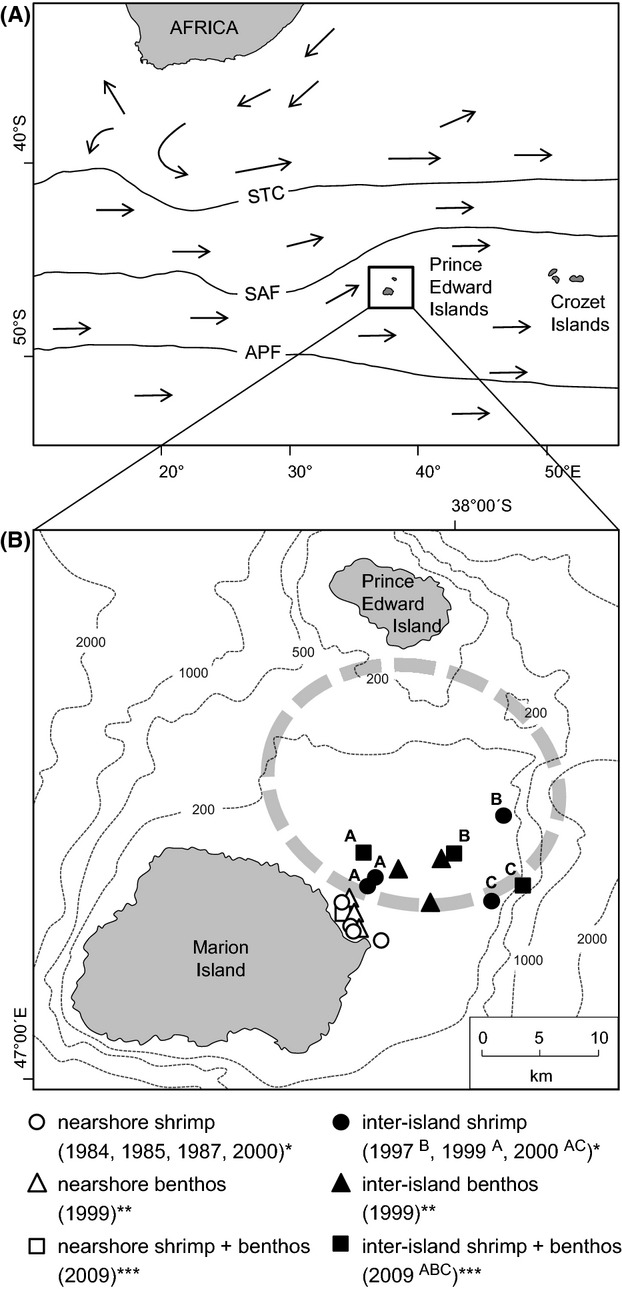
(A) Map of the Indian sector of the Southern Ocean indicating the average geographical position of the three major fontal systems and the position of the Prince Edward Islands (PEIs). STC, subtropical convergence; SAF, sub-Antarctic Front; APF, Antarctic Polar Front. (B) Location of sampling sites (separated into nearshore and inter-island sites) in the vicinity of the PEIs. Dashed oval indicates the region in which anticyclonic eddies form. Corresponding letters (A, B, and C) denote sample collections from different years but similar locations (see Fig. 3). *Shrimp (*Nauticaris marionis*) data obtained from Pakhomov et al. (2004); **benthos data obtained from Kaehler et al. (2000); ***shrimp and benthos data obtained from current study.

The PEIs are situated between the two major oceanic frontal systems that delineate the ACC: the sub-Antarctic Front (SAF) to the north and the Antarctic Polar Front (APF) to the south (Fig. 1), lying in the transition zone known as the Polar Frontal Zone (PFZ). The latitudinal position of the SAF plays a critical role in determining the oceanographic conditions around the islands (Perissinotto and Duncombe Rae 1990; Perissinotto et al. 2000; Ansorge and Lutjeharms 2002). When the SAF is far to the north of the PEIs, the flow rate of the ACC around the islands is slow enough that frictional forces between the current and the islands result in the formation of anticyclonic eddies that become trapped in the shallow region between the islands and in their lee (Perissinotto and Duncombe Rae 1990; Ansorge and Lutjeharms 2002). Freshwater runoff from the islands transports macronutrients, particularly reduced forms of nitrogen produced by top predators, into the surrounding waters (Ismail 1990; Perissinotto and Duncombe Rae 1990). The retention of freshwater runoff in the shelf waters also results in increased water column stability, as the depth of the mixed layer becomes shallower than the euphotic zone, thus promoting the development of dense phytoplankton blooms dominated by large diatoms (>20 μm) between and downstream of the islands, a phenomenon known as the “island mass effect” (Boden 1988; Perissinotto and Duncombe Rae 1990). This freshwater input, however, needs to be retained between the islands for a minimum of 15 days to enable the formation of these blooms (Perissinotto and Duncombe Rae 1990). During these blooms the bulk of the phytoplankton production is not utilized directly, and a large portion sinks to the seafloor, thus providing an important food source for the benthic community in the shallow shelf region of the islands (Perissinotto 1992; Pakhomov and Froneman 1999). In contrast, when the SAF lies farther south and close to the islands, the region around the PEIs experiences much higher flow rates, resulting in a flow-through system that prevents the formation of eddies and hence the development of phytoplankton blooms in close proximity to the islands (Perissinotto et al. 2000).

Despite their location in a generally low productivity region (Laubscher et al. 1993; Froneman et al. 2001), the PEIs support a diverse (∼550 species) and biomass-rich inter-island benthic community (Branch et al. 1993; Pakhomov and Froneman 1999) due to the enhanced productivity associated with bloom formation at these islands (Perissinotto 1992; Branch et al. 1993; Pakhomov and Froneman 1999; McQuaid and Froneman 2008). A key component of the benthos of the PEIs is the hyperbenthic shrimp *Nauticaris marionis*. Adult shrimps feed predominantly on benthic suspension- and deposit-feeders (Perissinotto and McQuaid 1990; Pakhomov et al. 1999), which are largely sustained by the autochthonous phytoplankton blooms (McQuaid and Froneman 2008; Allan 2011). In turn, these shrimp represent a dominant prey item in the diets of land-based predators such as gentoo penguins and Crozet shags that feed primarily within the inshore waters (Adams and Wilson 1987; Espitalier-Noël et al. 1988; Adams and Klages 1989; Crawford et al. 2003a). These shrimp contribute on average 26% and 25% by mass to the annual food consumed by gentoo penguins and Crozet shags, respectively (Espitalier-Noël et al. 1988; Adams and Klages 1989). It is important to note that their dietary contribution appears to vary seasonally, for example, from April to September their contribution ranges between 27–71% and 28–51% by mass (gentoo penguins and Crozet shags, respectively; Espitalier-Noël et al. 1988; Adams and Klages 1989). Furthermore, these shrimp periodically form an important food source for land-based predators that feed within both the inshore and offshore waters such as rockhopper and macaroni penguins (mixed-feeders; Brown and Klages 1987). During chick rearing rockhopper penguins, and to a lesser extent macaroni penguins, predominantly feed inshore (Brown 1987; Brown and Klages 1987). As a result, *N. marionis* forms an important trophic link between the sessile benthos and certain island-based predators (Perissinotto and McQuaid 1990).

It is becoming increasingly apparent that global climate change is affecting the terrestrial ecosystem of the PEIs through increased air temperatures, decreased precipitation and melting of the ice plateau (Smith 2002; Sumner et al. 2004; le Roux and McGeoch 2008). Evidence for the impacts of climate change on the marine ecosystem in the immediate vicinity of the PEIs is, however, generally lacking due to the difficulty in obtaining long-term data on marine organisms. Nonetheless, data from the vicinity of these islands indicate that the surface water temperatures have increased by >1°C since the 1950s (Mélice et al. 2003), and that this change in temperature has coincided with increased abundances of warm water zooplankton species over the past three decades (Pakhomov et al. 2000). Taken together, these trends suggest a southwards migration of the SAF, which is in agreement with recent studies that the strengthening and shifting of the southern hemisphere westerlies are concomitant to a poleward shift in the ACC (Oke and England 2004; Fyfe and Saenko 2005; Sokolov and Rintoul 2009) and SAF (Downes et al. 2011). As polar marine ecosystems are strongly shaped by large-scale physical processes, long-term shifts in the position of an oceanic front such as the SAF could feasibly have dramatic consequences for sub-Antarctic islands hundreds of kilometers away. We hypothesized that the long-term shift in the latitudinal position of the SAF has affected the primary productivity in the vicinity of the PEIs, due to increased occurrences of flow-through conditions, resulting in a temporal depletion in δ^13^C values of benthic organisms inhabiting the shallow shelf region of the islands.

## Materials and Methods

### Sample collection

This study was conducted aboard the MV *SA Agulhas* in April 2009 in the vicinity of the sub-Antarctic PEIs (46.77°S, 37.85°E), and forms part of the larger project entitled “Variability in Southern Ocean ecosystems” (contributing to the South African National Antarctic Programme). The data collected in 2009 were compared with published data (Kaehler et al. 2000; Pakhomov et al. 2004) to determine temporal variations in the isotopic signatures of the benthic, hyperbenthic, and pelagic communities in the vicinity of the PEIs.

The benthic and hyperbenthic communities were sampled in two regions: the nearshore region of Marion Island (<3 km from the shoreline) and the inter-island shelf region (>3 km; shelf between the islands; Fig. 1). The benthic and hyperbenthic organisms were collected (100–170 m depths) using a benthic trawl (mouth size: 100 × 30 cm), while pelagic samples (zooplankton) were collected at night (vertical tows; 100–140 m depths) over the inter-island shelf region using a WP-2 net (mouth area: 0.25 m^2^, mesh size: 200 μm). Following collections at each site, the benthic (suspension-feeders and scavengers/predators), hyperbenthic (almost exclusively dominated by the shrimp *N. marionis*), and pelagic organisms were separated according to feeding guilds to prevent postcapture feeding. The organisms were then left in GF/F filtered seawater (pore size 0.7 μm) for at least 12-h to facilitate gut evacuation. All samples were stored at −20°C for the duration of the cruise, and transferred to −80°C on return to the laboratory. In the laboratory, the frozen benthic invertebrates, hyperbenthic shrimps, and pelagic zooplankton were dissected (when possible) and muscle tissues separated for the analyses. Individuals were identified to species level using taxonomic keys, with the exception of sponges which were thus treated as a single taxonomic group. For small organisms such as the brachiopods several individuals were pooled to obtain an adequate signal. With the exception of the hydrozoan (only one individual collected), two to three replicate samples were prepared for analysis.

### Stable isotope analysis

The benthic, hyperbenthic, and pelagic samples were lyophilized at −60°C for 24-h. All samples were individually homogenized using a mortar and pestle. Brachiopods and echinoderms were treated with 1 M HCl to remove carbonates, rinsed in distilled water, and redried for 24-h at 60°C (Fry 1988; Fry and Wainright 1991). Samples were processed to obtain stable carbon and nitrogen isotope data (IsoEnvironmental cc, Department of Botany, Rhodes University) using a Europa Scientific Elemental Analyser coupled to a 20-20 Isotope Ratio Mass Spectrometer. In-house (beet sugar and ammonium sulfate) and certified (Casein) standards, calibrated against International Atomic Energy Agency standards (IAEA-CH-6 and IAEA-N-1), were used as internal calibrants. Isotope results were expressed as: δ (‰) = (R_sample_/R_standard_ − 1) × 1000, where δ (‰) is δ^13^C or δ^15^N and R_sample_ and R_standard_ are the ^13^C/^12^C or ^15^N/^14^N ratios of the sample and standard, respectively. As lipids are isotopically lighter than other biochemical components, potential variation in isotope signatures arising from variable lipid content among species was minimized by incorporating a correction model from Post et al. (2007): δ^13^C_normalized_ = δ^13^C_untreated_ − 3.32 + 0.99 (C:N), where C:N is the ratio of carbon-to-nitrogen. In addition, lipids were extracted (chloroform, methanol, and water, 2:1:0.8, v/v/v; Bligh and Dyer 1959) from a subset of samples to ensure corrected results were accurate.

### Temporal variations in stable isotope signatures

The stable carbon and nitrogen signatures of the hyperbenthic shrimp *N. marionis* (defatted samples) collected from 1984 to 2000 (*n* = 2–9) were obtained from Pakhomov et al. (2004). Stable carbon and nitrogen signatures for selected components of the benthic (nearshore and inter-island) and pelagic communities (defatted samples) from 1999 (*n* = 1–3) were obtained from Kaehler et al. (2000). The isotope signatures of nine benthic invertebrate species (sponge unknown species, hydrozoan *Plumularia insignis*; brachiopod *Magellania kerguelenensis*; polychaete *Thelepus extensus*; ophiuroids *Ophionotus hexactis* and *Ophiacantha vivipara*; asteroids *Porania antarctica* and *Odonaster meridionalis*) and two species of zooplankton collected from the vicinity of the islands (chaetognath *Sagitta gazellae*; euphausiid *Euphausia vallentini*) were compared between 1999 and 2009. Due to the limited data *t*-tests were used with an alpha level of 0.05.

The benthic invertebrates and shrimp *N. marionis* differ isotopically with region sampled (i.e., nearshore vs. inter-island; Kaehler et al. 2000; Allan 2011), therefore only animals collected from the same locations within the nearshore and inter-island regions were compared between studies to ensure compatibility of the data (Fig. 1). For the shrimp, individuals were divided into one of five size classes according to carapace length (4.0–5.0 mm, 5.5–6.5 mm, 7.0–8.0 mm, 8.5–9.5 mm, and 10.0–11.0 mm), measured from the posterior margin of the orbit to the dorsal midline of the posterior margin of the carapace (Pakhomov et al. 1999). As isotopic signatures of *N. marionis* differed among these size classes (Allan 2011), only shrimps within the same size class as those analyzed in Pakhomov et al. (2004), carapace length 5.5–6.5 mm, were included. Unlike *N. marionis*, the benthic and pelagic organisms did not show variation in isotopic signatures among size classes (Allan 2011). All animals were collected during April/May (1984–2009) using the same sampling gear and were defatted prior to isotope analysis. The δ^13^C and δ^15^N data were all obtained from fresh tissues, with the exception of the isotope signatures of *N. marionis* from 1984, 1985, 1987, and 1997, which were corrected for preservation according to the correction factor calculated by Pakhomov et al. (2004).

### Trends in top predator populations

Population trends for the land-based top predators at the PEIs (populations on Marion Island) were obtained from previous population studies (Hofmeyr et al. 2006; Crawford et al. 2009a,b; Ryan et al. 2009). We divided the predators into those that feed primarily inshore (inshore-feeders: gentoo penguin *Pygoscelis papua*; Crozet shag *Phalacrocorax* [*atriceps*] *melanogenis*), those that feed inshore and offshore (seasonally variable mixed-feeders: southern [eastern] rockhopper penguin *Eudyptes chrysocome* [*filholi*]; macaroni penguin *Eudyptes chrysolophus*), and those that feed primarily farther offshore (offshore-feeders in the vicinity of frontal systems: king penguin *Aptenodytes patagonicus*; dark-mantled sooty albatross *Phoebetria fusca*; light-mantled sooty albatross *Phoebetria palpebrata*; wandering albatross *Diomedea exulans*; grey-headed albatross *Thalassarche chrysostoma*; southern giant petrel *Macronectes giganteus*; northern giant petrel *Macronectes halli*; sub-Antarctic fur seal *Arctocephalus tropicalis*; Antarctic fur seal *Arctocephalus gazella*).

## Results

### Temporal variation in stable isotope signatures

**Figure 2:**
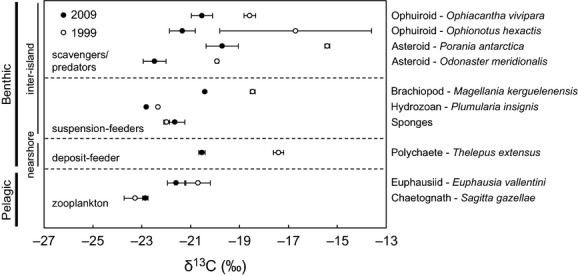
Temporal shift in δ^13^C signatures (‰) of pelagic and benthic invertebrates at the Prince Edward Islands between 1999 and 2009. Pelagic samples (zooplankton) were collected from the inter-island shelf waters, while benthic samples were collected from the nearshore and inter-island regions. Pelagic and benthic data: April 1999 (obtained from Kaehler et al. 2000); April 2009 (current study). Error bars represent standard deviations.

The δ^13^C signatures of the benthic organisms collected in 2009 were significantly more depleted than those collected in 1999 (paired *t*-test [df = 4]: *P* < 0.05), while the zooplankton (euphausiids and chaetognaths) and sponges had similar isotope signatures during both studies (paired *t*-test [df = 4]: *P* > 0.05; Fig. 2). The notable exceptions were hydrozoans and brachiopods which only consisted of one individual. The inter-island hydrozoan and brachiopod had a δ^13^C depletion of 0.48 and 1.96, respectively. The nearshore polychaete had a mean δ^13^C depletion of 3.14‰, while the inter-island ophiuroids *Ophiacantha vivipara* and *Ophionotus hexactis*, and the inter-island asteroids *Odonaster meridionalis* and *Porania antarctica* had a mean δ^13^C depletion of 1.96‰, 4.64‰, 2.56‰, and 4.31‰, respectively.

**Figure 3:**
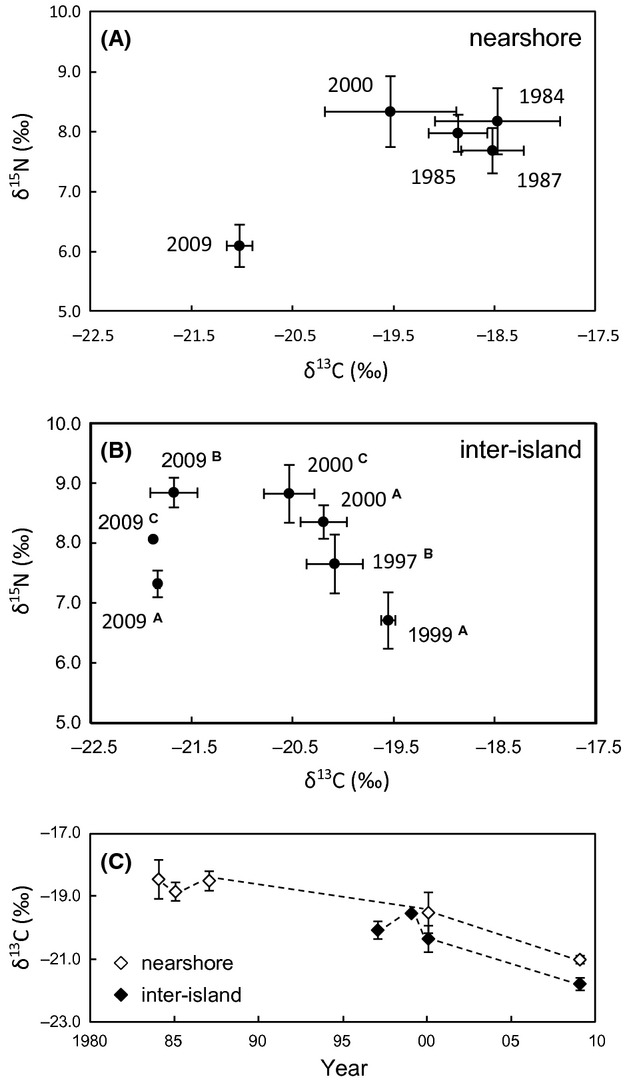
δ^13^C and δ^15^N signatures (‰) of *Nauticaris marionis* from 1984 to 2009 at the Prince Edward Islands in the (A) nearshore and (B) inter-island regions. (C) Temporal shifts in δ^13^C signatures (‰) of *N. marionis* in the nearshore and inter-island regions over the last three decades. *Nauticaris marionis* data: April/May 1984, 1985, 1987, 1997, 1999, and 2000 (obtained from Pakhomov et al. 2004); April 2009 (current study). Corresponding letters (A, B, and C) denote sample collections from different years but similar locations in the inter-island region. Error bars represent standard deviations.

*Nauticaris marionis* collected in 2009, from both the nearshore and inter-island regions, were more ^13^C-depleted compared to shrimps collected in previous years (1984–2000; Fig. 3A–C). Between 2000 and 2009 the nearshore and inter-island shrimps had a mean δ^13^C depletion of 1.43‰ and 1.49‰, respectively. While there was some inter-annual variation in the δ^13^C signatures of *N. marionis* among years, the overall trend was a marked depletion in δ^13^C values over time in both the nearshore and inter-island individuals (Fig. 3A–C). Over the period 1984 to 2009 the nearshore shrimps had a δ^13^C depletion rate of 0.10‰ year^−1^, while the inter-island shrimps had a δ^13^C depletion rate of 0.14‰ year^−1^ over the period 1997 to 2009.

### Trends in top predator populations

Predator population trends were calculated as proportional changes in the predator population sizes to allow comparisons among the different species. Therefore, the maximum population size recorded is 100% and all lower values indicate smaller populations. Note that the population sizes for king penguins, sub-Antarctic fur seals, and Antarctic fur seals were determined from the number of chicks/pups, while the population sizes for all other predators (shags, penguins, albatrosses, and petrels) were determined from breeding pairs.

**Figure 4:**
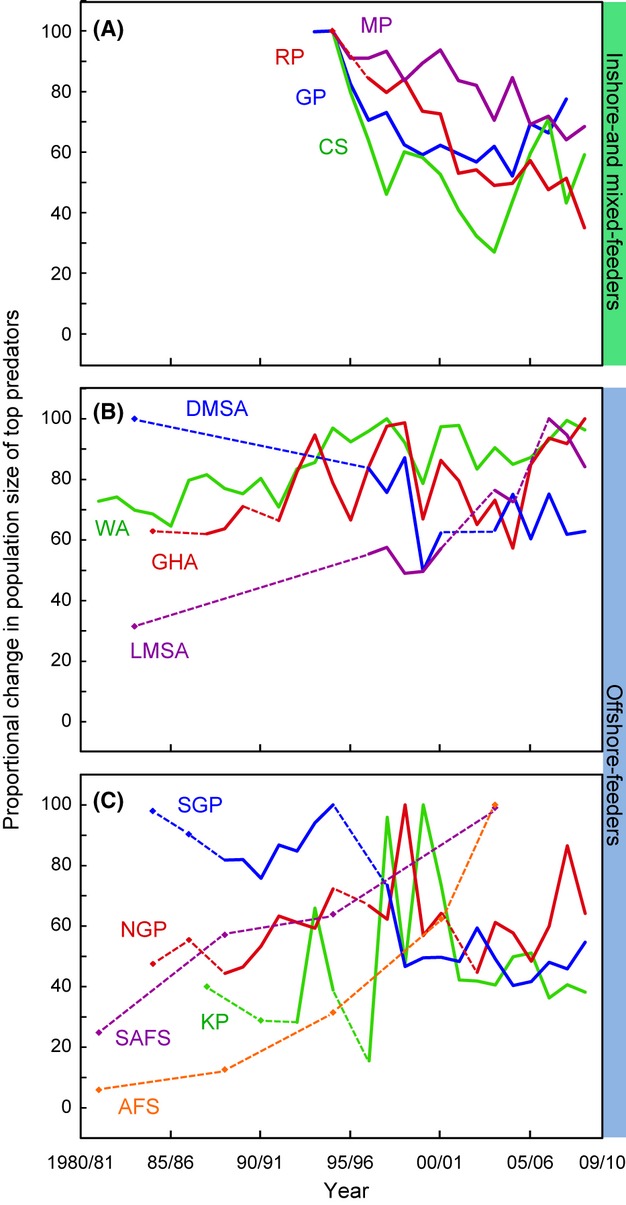
Proportional changes in the population sizes of land-based predators at the Prince Edward Islands (Marion Island populations) over the last three decades: (A) inshore- and mixed- (inshore and offshore) feeders (obtained from Crawford et al. 2009a,b); and (B, C) offshore-feeders (obtained from Hofmeyr et al. 2006; Ryan et al. 2009). CS, Crozet shags; GP, gentoo penguins; RP, rockhopper penguins; MP, macaroni penguins; KP, king penguins; WA, wandering albatrosses; GHA, grey-headed albatrosses; DMSA, dark-mantled sooty albatrosses; LMSA, light-mantled sooty albatrosses; SGP, southern giant petrels; NGP, northern giant petrels; SAFS, sub-Antarctic fur seals; AFS, Antarctic fur seals.

Over the last two decades there have been overall decreases in the population sizes of inshore-feeding predators such as gentoo penguins and Crozet shags, however, these populations have begun to show an increase since the mid 2000s due to improved breeding success (1994/95–2008/09, polynomial regression: *n* = 15, *r* = 0.950, *P* < 0.001, and *n* = 15, *r* = 0.815, *P* < 0.001, respectively; Crawford et al. 2009a,b; Fig. 4A). Prior to the recent population increase, the gentoo penguin and Crozet shag populations decreased in size by approximately 4.8% and 8.1% year^−1^, respectively. The rockhopper and macaroni penguins, which feed in both the inshore and offshore regions (mixed-feeders), also experienced decreases in population sizes over the same period (1994/95–2008/09, linear regression: *n* = 14, *r* = 0.932, *P* < 0.001, and *n* = 15, *r* = 0.888, *P* < 0.001, respectively; Crawford et al. 2009b; Fig. 4A). The rockhopper penguin populations showed a similar decrease (∼4.6% year^−1^) to that of the inshore-feeding gentoo penguins, while the macaroni penguins have shown less of a decline in population size (∼2.8% year^−1^). It is important to note that, unlike the inshore-feeders, the rockhopper, and macaroni penguin populations have continued to decline to date.

For the land-based offshore-feeding predators, there have been overall increases in the population sizes of the light-mantled sooty albatrosses and wandering albatrosses over the last two to three decades (linear regression slope: 1.052 ± 0.014, *P* < 0.01 [1996/97–2008/09], and 1.012 ± 0.001, *P* < 0.01 [1981/82–2008/09], respectively; Ryan et al. 2009; Fig. 4B). The sub-Antarctic fur seals and Antarctic fur seals have also experienced population increases following their protection during the 20th Century (Hofmeyr et al. 2006; Fig. 4C). Predators such as dark-mantled sooty albatrosses, however, have shown a significant decrease in population size since the mid 1990s (1996/97–2008/09 linear regression slope: 0.981 ± 0.009, *P* < 0.05), continuing the negative population trend observed since the early 1980s (Ryan et al. 2009; Fig. 4B). Other predators such as southern giant petrels have experienced a decrease in breeding pairs from the 1980s to the late 1990s, which has since stabilized (1998/99 onwards; Ryan et al. 2009; Fig. 4C). Some predator species such as the grey-headed albatross, northern giant petrel, and king penguin have displayed large annual fluctuations in population size but their populations have remained stable overall (Fig. 4B and C). For instance, grey-headed albatross population sizes have remained relatively constant since the 1990s with no significant trends since 1997/98 (Ryan et al. 2009). King penguin populations have also shown vast fluctuations in size, mostly due to their interannual variability in chick survival, but have remained stable overall (Crawford et al. 2009b). In addition, the numbers of northern giant petrels breeding at Marion Island, apart from the two population peaks most likely reflecting higher counts due to greater search effort, have remained constant since 1996/97 (Ryan et al. 2009).

## Discussion

This study represents an analysis of the temporal trends in the isotopic signatures of the inshore benthos of the PEIs in order to identify the impact of regional climate change on this productive ecosystem and its potential implications for top predators that inhabit these islands.

Here, we present evidence of a measureable temporal shift in the carbon signatures of an inshore sub-Antarctic marine ecosystem. With the notable exceptions of the sponges and hydrozoans, the benthic invertebrates (suspension-feeders and scavengers/predators) inhabiting the shallow shelf region of the PEIs showed a significant depletion in their stable carbon isotope signatures (δ^13^C) from 1999 to 2009 (depletions ranged from 1.96‰ to 4.64‰; Fig. 2). The shrimp *N. marionis*, for which we have longer term data, too showed a depletion in δ^13^C values in both nearshore and inter-island individuals over the period 1984 to 2009 (Fig. 3A–C). Over the corresponding period 1999 to 2009, inter-island shrimps showed a depletion in carbon signatures of 2.28‰, which is within the range of that recorded in the inter-island benthos (δ^13^C depletion of between 1.96‰ and 4.64‰). In contrast, no such temporal depletions were apparent in the carbon isotope signatures of pelagic zooplankton collected at the PEIs (between 1999 to 2009; Fig. 2). As the zooplankton collected at the PEIs were transported there from the upstream region by the easterly flowing ACC, these animals would have been feeding in the oligotrophic open waters of the PFZ prior to their capture (Perissinotto and McQuaid 1992; McQuaid and Froneman 2008). The dissimilar temporal trends in isotopic signatures in pelagic zooplankton and benthic invertebrates (including the shrimp *N. marionis*) suggests that the gradual depletion of δ^13^C signatures in the benthos reflects a change in the biology of the primary producers that support the shallow shelf food web at the PEIs.

At the PEIs, phytoplankton form the dominant food source for the benthic community inhabiting the shallow shelf region in the vicinity of the islands (McQuaid and Froneman 2008; Allan 2011). Variations in δ^13^C signatures of phytoplankton have been linked with changes in isotopic fractionation due to alterations in water temperature, molecular CO_2_ concentrations, and growth conditions (Bidigare et al. 1997; Laws et al. 1997; Rau et al. 1997; Burkhardt et al. 1999). As oceanic δ^13^C values tend to become enriched with increasing temperature and depleted with increasing molecular CO_2_ concentrations (Rau et al. 1997), increased atmospheric CO_2_ resulting from the burning of fossil fuels could account for some of the observed depletion in δ^13^C signatures. In sub-polar regions, a depletion of ∼0.015‰ year^−1^ has been calculated in the δ^13^C signatures of oceanic dissolved inorganic carbon (Suess effect; McNeil et al. 2001). Between 1984 and 2009, the Suess effect would account for only a 0.38‰ depletion in the δ^13^C signatures of organic matter, a value substantially smaller than the 2.55‰ depletion observed in the shrimps over this period. As a result, anthropogenic CO_2_ cannot be the sole driver of the variations in δ^13^C signatures observed in the *N. marionis* population. Phytoplankton growth rates also have a strong negative relationship with carbon isotope fractionation, with rapidly growing populations having more enriched δ^13^C values than slow growing forms (Bidigare et al. 1997; Laws et al. 1997; Burkhardt et al. 1999). Dense, rapidly growing phytoplankton communities associated with the “island mass effect” have enriched δ^13^C values compared to those in the less productive open waters of the PFZ in which the islands lie (∼−23.3‰ vs. ∼−24.8‰, respectively; Kaehler et al. 2000). Thus, the temporal depletion of δ^13^C signatures observed in the shrimp and various benthic invertebrates likely reflects a long-term decline of enhanced primary production (frequency of diatom blooms) in the vicinity of the islands.

The sensitivity of the ACC to climate change, in particular to the strengthening and shifting of the westerly wind belt (Oke and England 2004), has been of great interest. Climate models run under global warming conditions suggest that these wind stress changes are concomitant to a poleward shift in the ACC (Oke and England 2004; Fyfe and Saenko 2005; Sokolov and Rintoul 2009) and the three branches of the SAF (Downes et al. 2011). This southward migration of the SAF (indirect effect of climate change) seems to have contributed to a long-term decline in the frequency of phytoplankton blooms (“island mass effect”) at the PEIs, reflected by temporal depletions in the carbon isotope signatures of the various benthic invertebrates that inhabit the shelf region of the islands. The absence of a trend in the isotopic signatures of the sponges and hydrozoans supports this interpretation, as these two taxa showed dissimilar feeding behaviors from the other benthic species. Sponges generally consume small particles in the pico- and nano-size range (<20 μm; Reiswig 1990; van de Vyver et al. 1990; Pile et al. 1996) and are unlikely to feed on phytoplankton blooms consisting of large diatoms, so should be unaffected by shifting frequencies of phytoplankton blooms driven by larger diatoms (>20 μm). The hydrozoans at the PEIs appear to obtain a large proportion of their diet from animal prey such as zooplankton (elevated proportions of the fatty acid 18:1ω9 [>14.0%] and a carnivory index [18:1ω9/18:1ω7] >3.0; Allan 2011). As such, the hydrozoans, like the sponges, are unlikely to utilize large amounts of diatom production or respond directly to changes in the frequency of diatom blooms. As phytoplankton are an important food source for the food webs at the PEIs (Kaehler et al. 2000; Allan 2011), decreased occurrences of diatom blooms between the islands will have strong effects on higher trophic levels.

The PEIs seasonally support up to five million land-based predators that come to the islands to breed and molt (Williams et al. 1979; Condy 1981; Ryan and Bester 2008). Over the last three decades, there has been an overall decrease in the population sizes of predators that predominantly feed inshore such as gentoo penguins, Crozet shags, and rockhopper penguins (Fig. 4A; Crawford et al. 2003b, 2009a,b); while species that feed regularly in both the inshore and offshore regions, such as macaroni penguins, have shown smaller declines in population size over the same period (Fig. 4A; Crawford et al. 2003b, 2009b). Conversely, population sizes of offshore-feeding land-based predators (light-mantled sooty albatrosses, wandering albatrosses, grey-headed albatrosses, northern giant petrels, southern giant petrels, sub-Antarctic fur seals, and Antarctic fur seals) have been stable or increasing over the corresponding period, with the exception of dark-mantled sooty albatrosses (Fig. 4B and C; Hofmeyr et al. 2006; Ryan et al. 2009). Top predator populations are influenced by a variety of direct and indirect factors that include both present population pressures and the relaxation of earlier pressures. Competition both within and among species for resources such as food and space for breeding can be important as populations increase. Fisheries can have direct effects through mortality of animals as by-catch, while enhanced mitigation measures can be followed by population recovery from this effect. Fisheries can also remove prey species, again either directly or indirectly as by-catch. Man can have even more direct effects by targeting the land-based predators themselves and again these populations can show recovery once exploitation ceases. Finally, changing environmental conditions due to the effects of climate change have been suggested as having direct effects on populations, for example, increased wind speeds have been linked to population increases of wandering albatrosses due to improved fitness, foraging performance, and breeding success (Weimerskirch et al. 2012). Changes in predator populations at the PEIs are likely a result of a combination of several of these factors. As the offshore-feeding top predators at the PEIs forage over great distances from the islands, they are not as reliant on local productivity in the vicinity of the islands as the inshore-feeding top predators which we will focus on.

Inshore-feeders (gentoo penguins and Crozet shags) at the PEIs are pursuit-divers that feed mainly on demersal fish (Nototheniidae) and *N. marionis* (Adams and Wilson 1987; Espitalier-Noël et al. 1988; Adams and Klages 1989; Crawford et al. 2003a), whereas the mixed-feeders (rockhopper and macaroni penguins) feed primarily on pelagic fish (in particular Myctophidae) and crustaceans such as euphausiids and *N. marionis* (Brown and Klages 1987). Although industrial fishing at the PEIs (from the mid 1990s) has depleted demersal fish stocks dramatically (particularly those of *Lepidonotothen squamifrons* and *Dissostichus eleginoides*; Lombard et al. 2007), the continued population decreases of mixed-feeders that do not depend on these Nototheniidae fish species indicates that the decreases in inshore-feeding predator populations are not primarily driven by human exploitation of prey species. Fishing activities at the PEIs have been prohibited since December 2004 (Lombard et al. 2007), which is correlated with an apparent increase in the populations of fish-eating Crozet shags and gentoo penguins but not of mixed-feeders. The depletion of fish stocks, however, most likely resulted in a shift in the diet of the Crozet shags and gentoo penguins as *L. squamifrons*, their dominant dietary fish in the 1980s (Blankley 1981; Espitalier-Noël et al. 1988), was replaced by *Gobionotothen marionensis* and *Lepidonotothen larseni* by the late 1990s (Crozet shags; Crawford et al. 2003a). Conversely, the dietary contribution of *N. marionis* remained the same over this period (Espitalier-Noël et al. 1988; Crawford et al. 2003a).

*Nauticaris marionis*, which consume benthic suspension- and deposit-feeders (Perissinotto and McQuaid 1990; Pakhomov et al. 1999), represents an important food source for land-based predators that feed inshore (Brown and Klages 1987; Espitalier-Noël et al. 1988; Adams and Klages 1989). In addition, these shrimps together with benthic polychaetes are the dominant food sources for the fish *G. marionensis* (Bushula et al. 2005), which in turn is an important prey species to Crozet shags (Crawford et al. 2003a). As a result, the benthic and hyperbenthic communities of the shallow shelf region at the PEIs represent a crucial link between primary producers and certain top predators. Since the initial photographic assessments in the 1980s (Branch et al. 1993), there have been no additional quantitative studies on the biomass and abundance of the benthic community around the PEIs. Consequently, we cannot determine whether shifts in the regional hydrodynamics around the islands have resulted in changes in the biodiversity, abundance, and biomass of the benthos. Nevertheless, declines in the abundances of predators that rely heavily on prey inhabiting the inshore areas of the PEIs suggest changes in prey availability within this island ecosystem. Changes in prey availability are likely driven by a combination of local factors such as fisheries impacts on prey populations and changes in competitive interactions among predators for food. In addition to these local factors, our results indicate that changes in prey availability may also arise indirectly through the effects of regional climate change on the marine ecosystem at the PEIs. In this case, regional climate change has caused a southward shift in the position of a major current system (the SAF), which has affected the primary productivity in the vicinity of the PEIs (decreased frequency of diatom blooms) due to increased occurrences of flow-through conditions. The shift in dominant primary production at the PEIs from diatom-dominated communities to less productive small cell-dominated phytoplankton communities is reflected isotopically in the primary consumers (benthic suspension- and deposit-feeders) inhabiting the inshore region of the islands. In addition, the shift in primary producers consumed at the base of the food web is also apparent in higher trophic levels such as ophuiroids, asteroids, and the shrimp *N. marionis*, that inhabit the inshore region. These results indicate a fundamental shift in the balance between allochthonous and autochthonous trophic pathways within the PEI region, thus highlighting the vulnerability of marine ecosystems to large-scale changes in physical conditions. Most importantly, these results indicate that the consequences of climate change may be indirect.

Our results demonstrate that the most powerful effects of climate change on natural ecosystems may be obscured because they are indirect, thus making it difficult to detect cause and effect relationships. This is particularly problematic where appropriate long-term data (such as biodiversity, abundance, and biomass of benthic communities) are generally lacking. Indirect effects of climate change, such as those observed at the PEIs, coupled with the lack of long-term data for key marine ecosystem components highlight the concerns of the International Program on the State of the Ocean (IPSO) that cumulative impacts on the ocean are greater than previously thought (Rogers and Laffoley 2011).
